# Supplementary material for: Integrative genome-wide analysis reveals the role of WIP proteins in inhibition of growth and development
Source: Commun Biol. 2020 May 15;3:239. doi: 10.1038/s42003-020-0969-2 (PMC7229033; doi:10.1038/s42003-020-0969-2)
Supplement: Supplementary file 1 — Supplementary Information [file 42003_2020_969_MOESM1_ESM.pdf]

SUPPLEMENTARY FIGURES

Integrative genome-wide analysis reveals the role of WIP proteins in inhibition of growth and development

Maria Victoria Gomez Roldan[1,+], Farhaj Izhaq[1,+], Marion Verdenaud[1,2], John Eleblu[1], Aimen Haraghi[1], Vivien Sommard[1], Pierre Chambrier[2], David Latrasse[1], Teddy Jegu[1], Moussa Benhamed[1], Judit Szécsi[2], Mohammed Bendahmane[2], Adnane Boualem[1], Abdelhafid Bendahmane[1,*]

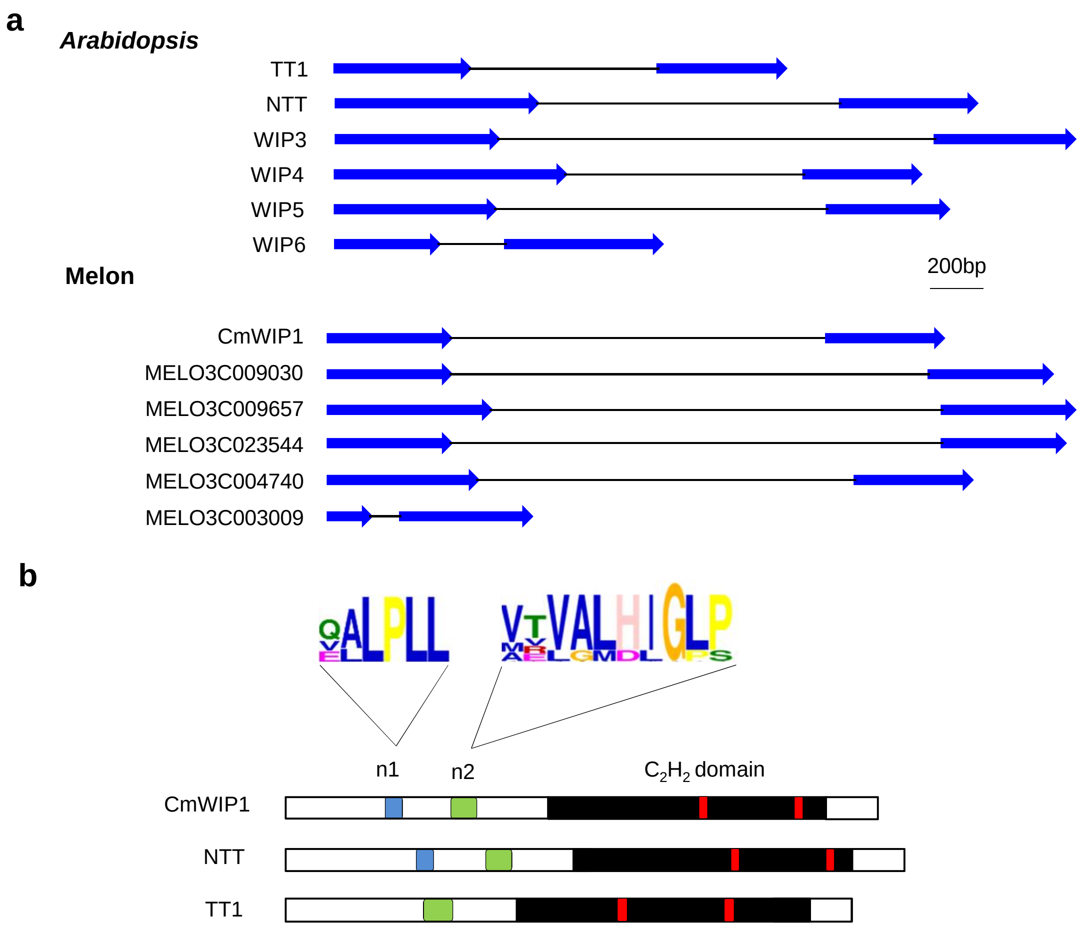

Supplementary Figure 1. Sequences analysis of Arabidopsis and melon WIP genes. a) Structure of Arabidopsis and melon WIP genes. All of them contain two exons. **b**) Schematic representation of melon (CmWIP1) and Arabidopsis (TT1 and NTT) proteins showing the N-terminal part with two conserved domains (n1 and n2) and the conserved C-terminal part with two nuclear localization signals (NLSs, red).

TT1-AT1G34790

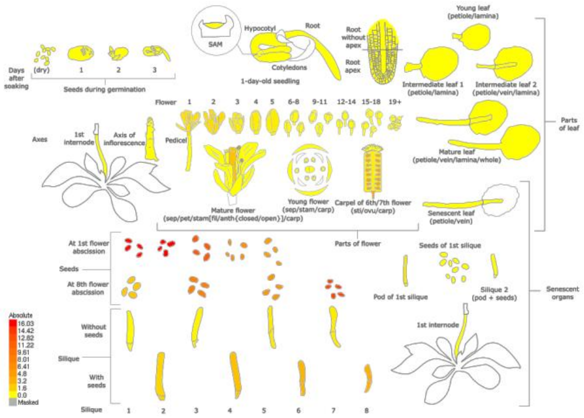

NTT-AT3G57670

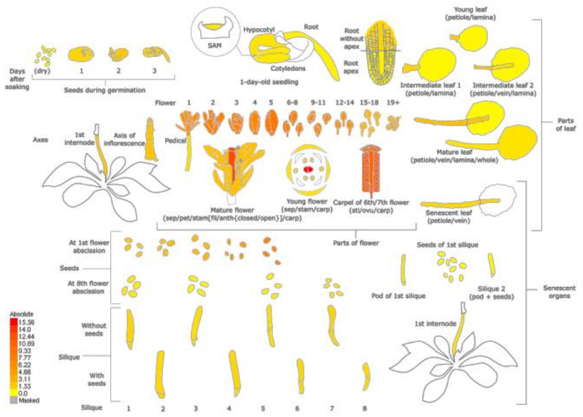

WIP3-AT1G08290

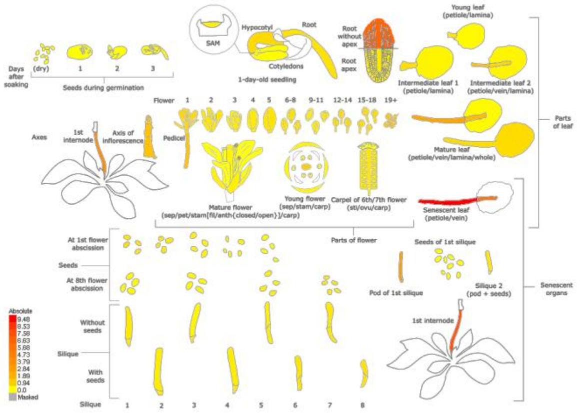

WIP4-AT3G20880

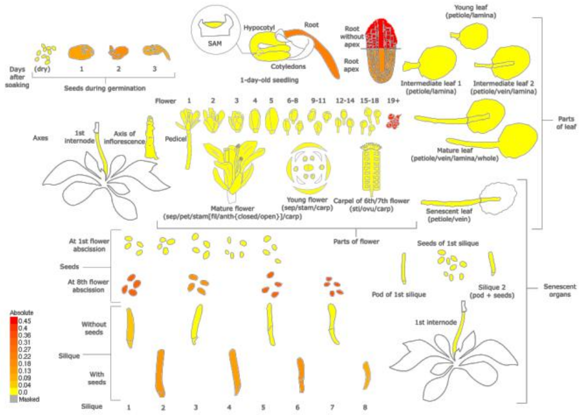

WIP5-AT1G51220

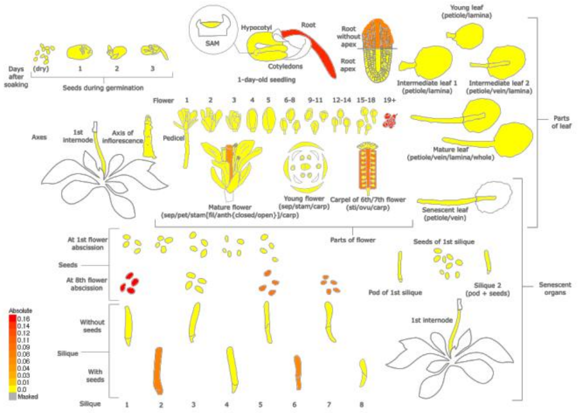

WIP6-AT1G13290

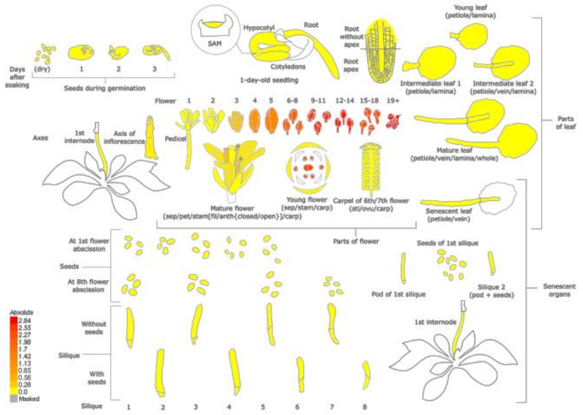

Supplementary Figure 2. Expression patterns of the six WIP Transcription Factors in *Arabidopsis thaliana.* A high resolution map of the *A. thaliana* developmental transcriptome based on RNA-seq profiling of different tissues/organs (base on BAR eFP Browser, Klepikova Atlas, http://bar.utoronto.ca/).

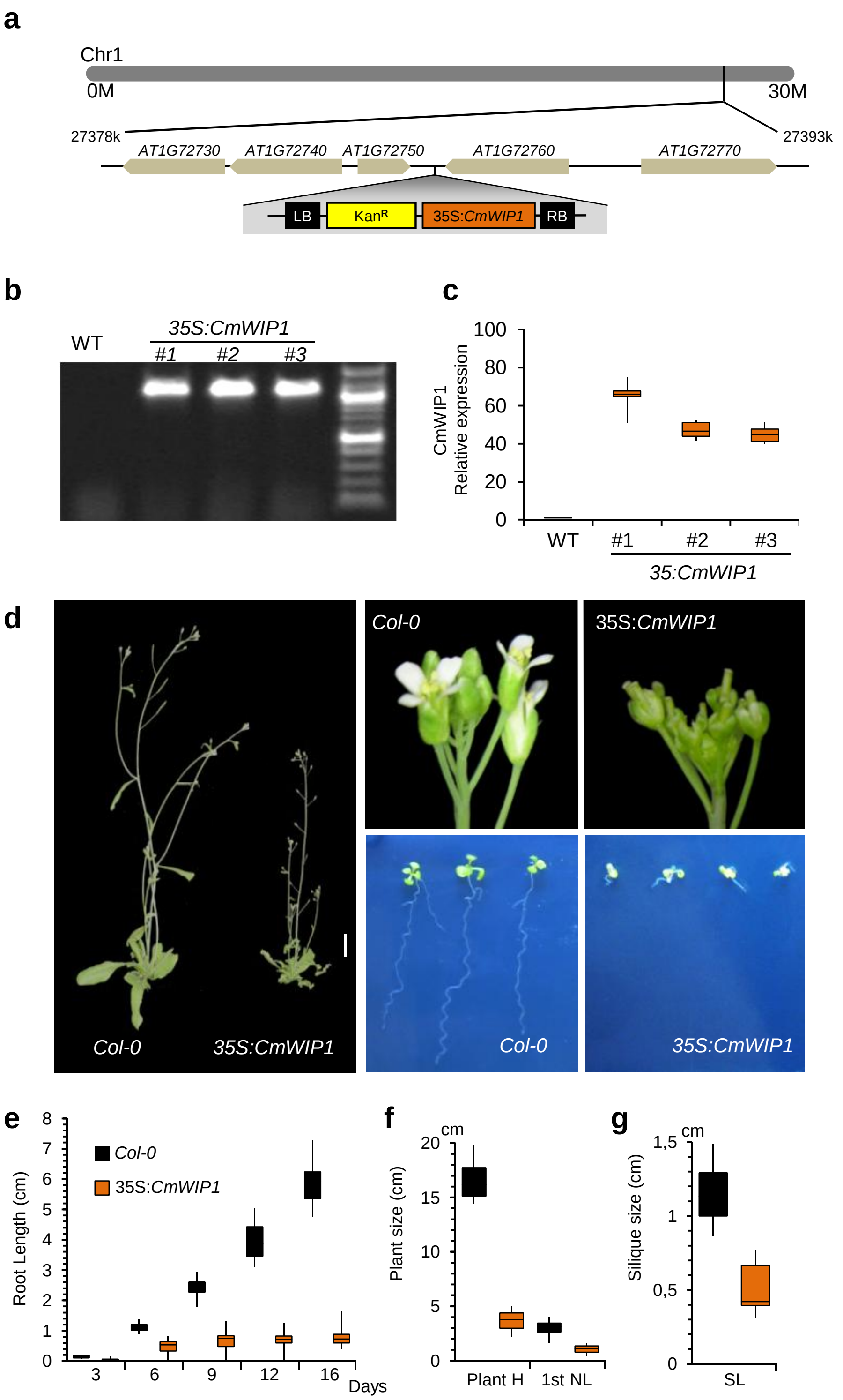

Supplementary Figure 3. Phenotypic characterization of *Arabidopsis 35S:CmWIP1* transgenic lines. a) Schematic representation of the *35S:CmWIP1* insertion site in *Arabidopsis* genome, determined by the sequencing of the flanking sequence tag (FST). **b**) PCR analysis validating the presence of the *35S:CmWIP1* T-DNA. 100bp DNA leader used as size marker. **c**) RT-qPCR expression analysis of *CmWIP1* in *Col-0* and in three *35S:CmWIP1* transgenic lines (*n*=6 biologically independent plants). **d**) Representative mature plants, of *35S:CmWIP1* transgenic lines, showing reduced plant size in general, compared to Col-0 untransformed control. Inflorescence morphology of *Col-0* and *35S:CmWIP1* transgenic lines showing whorl defects affecting plant fertility. Root phenotype of 6-days old seedlings of WT *Col-0* and *35S:CmWIP1* transgenic lines grown on MS agar medium. Quantification in *Col-0* and *35S:CmWIP1* transgenic lines of **e**) root length (*n*=18 biologically independent plants), **f**) general plant size (*n*=4 biologically independent plants) and **g**) silique size (*n*=24, 8 siliques on 3 biologically independent plants). Plant H=height, 1stNL=first node height, and SL=silique.

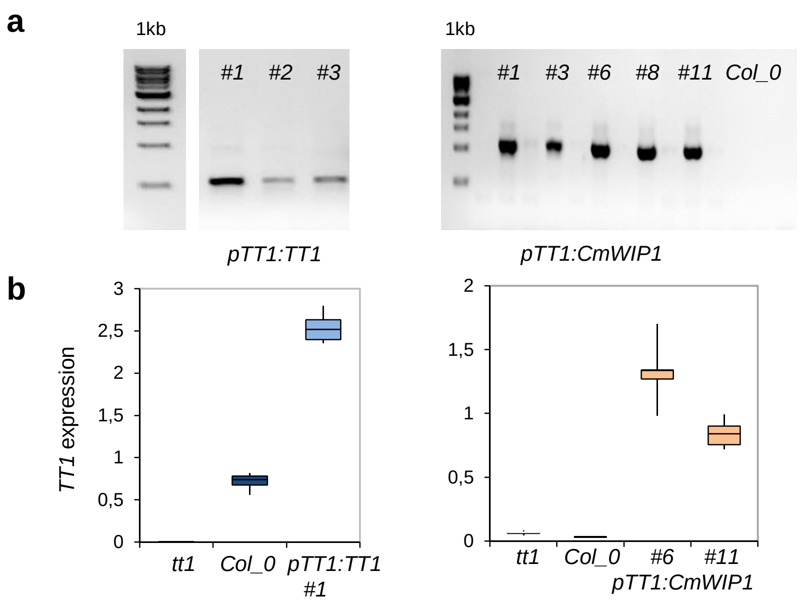

Supplementary Figure 4. Analysis of Arabidopsis *tt1* mutant transgenic plants expressing *CmWIP1* coding sequence under the control of *TT1* promoter. **a**) Analysis of presence of *pTT1:TT1* and *pTT1:CmWIP1* T-DNAs by PCR in independent transgenic lines. Amplification fragments were run in a gel with a 1kb ladder marker. **b**) RT-qPCR analysis of *TT1* and *CmWIP1* transcript in *pTT1:TT1* and *pTT1:CmWIP1* transgenic lines. No expression of *CmWIP1* in WT (*Col-0*) and *tt1* plants was detected.

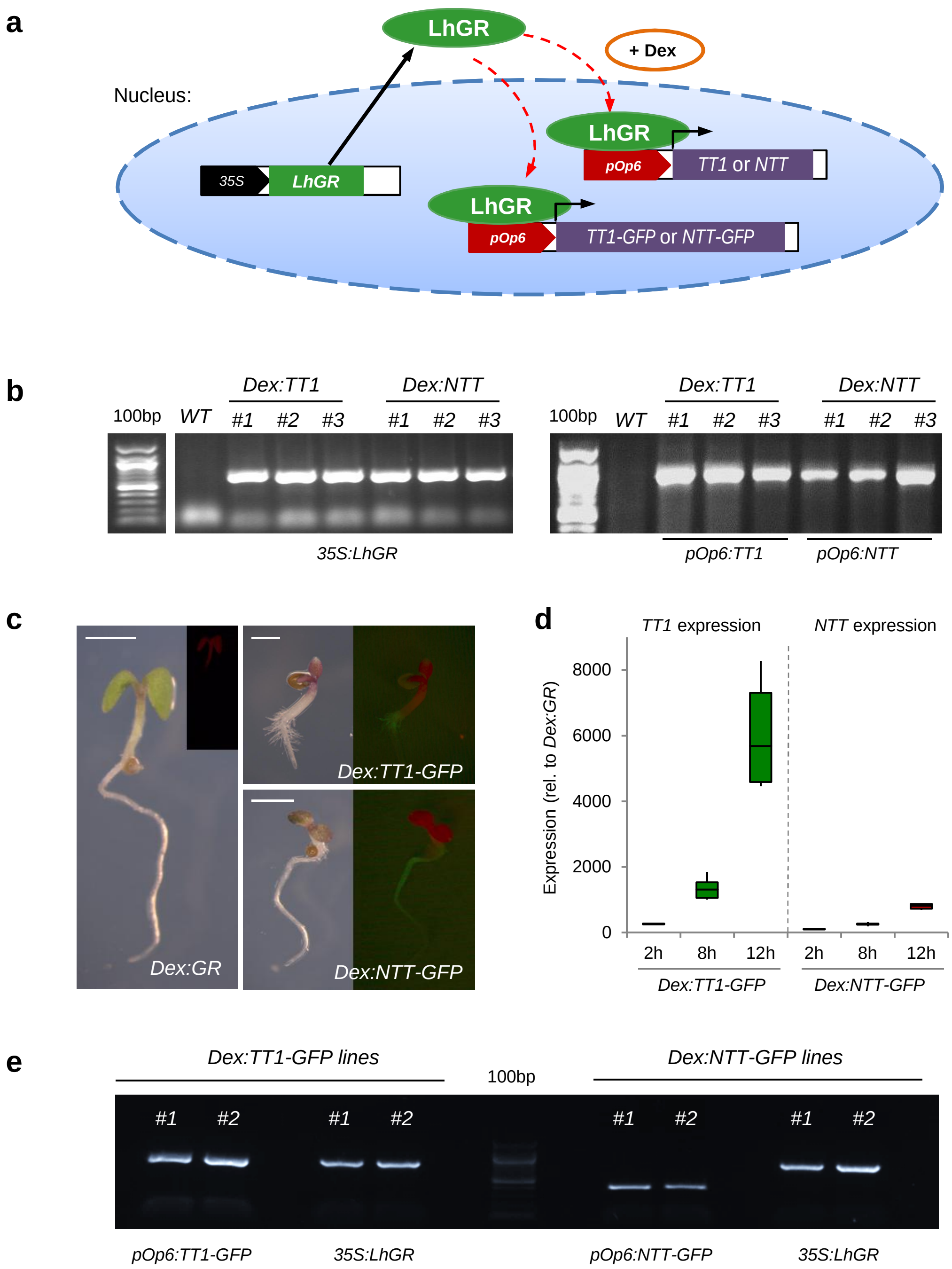

Supplementary Figure 5. The dexamethasone-inducible system used to overexpress *TT1* and *NTT* during seedling development. **a**) The glucocorticoid binding domain (*LhGR*) is constitutively expressed under the control of *CaMV 35S* promoter and in the presence of Dexamethasone (Dex) the LhGR will bind the pOp6 promoter to induce expression of *TT1* and *NTT* (with or without GFP-tag). **b**) Analysis of independent *Dex:TT1* and *Dex:NTT* transgenic lines for presence of the transgenes. **c**) Phenotype of five-days old representative transgenic lines (*Dex:GR*, *Dex:TT1-GFP* and *Dex:NTT-GFP*) grown on MS medium containing 1µM of Dex. Scale = 1 mm. **d**) Expression of *TT1* and *NTT* in *Dex:TT1-GFP* and *Dex:NTT-GFP* transgenic lines (related to *Dex:GR* control lines) in seven-days old seedlings induced for 2h, 8h and 12h with 1µM Dex. **e**) Analysis of independent *Dex:TT1-GFP* and *Dex:NTT-GFP* transgenic lines for presence of the transgenes. PCR fragments validating the presence of *LhGR*, *pOp6:TT1* and *pOp6:NTT or the* WIP fused to GFP T-DNAs are shown. 100bp DNA leader used as size marker.

a

Sample name	Raw reads	Aligned Reads	% aligned	Unique position	% unique
TT1-GFP	30,326,331	27,605,446	91,0	19,171,099	63,2
TT1 Input	28,482,764	28,254,104	99,2	18,065,276	63,4
NTT-GFP 1	26,446,147	15,071,778	57,0	10,535,902	39,8
NTT Input-1	35,040,996	33,814,119	96,5	16,716,265	64,7
NTT-GFP 2	46,170,449	38,101,307	82,5	28,982,615	76,1
NTT Input-2	32,730,724	31,889,888	97,4	15,230,594	46,5

b

	Dex:GR-1	*Dex:GR-2*	*Dex:TT1-1*	*Dex:TT1-2*	*Dex:GR-1*	*Dex:GR-2*	*Dex:NTT-1*	*Dex:NTT-2*
Reads number	49,212,027	53,432,070	33,984,996	47,303,199	47,871,092	38,087,650	40,099,458	30,281,994
% Mapped reads	94,8	94,0	94,6	94,0	95,6	95,8	95,5	95,2
% Reads with multiple alignments	3,0	3,1	2,7	2,8	3,1	3,2	2,7	2,5

Supplementary Figure 6. ChIP-seq and RNA-seq experiments. a) Number of ChIP-seq reads on Dex:*TT1-GFP* and Dex:*NTT-GFP* data and its corresponding input aligned to the *Arabidopsis* TAIR10 reference genome. **b**) Number of RNA-seq reads on Dex:*TT1* and Dex:*NTT* data and its corresponding control *Dex:GR* lines aligned to the *Arabidopsis* TAIR10 reference genome.

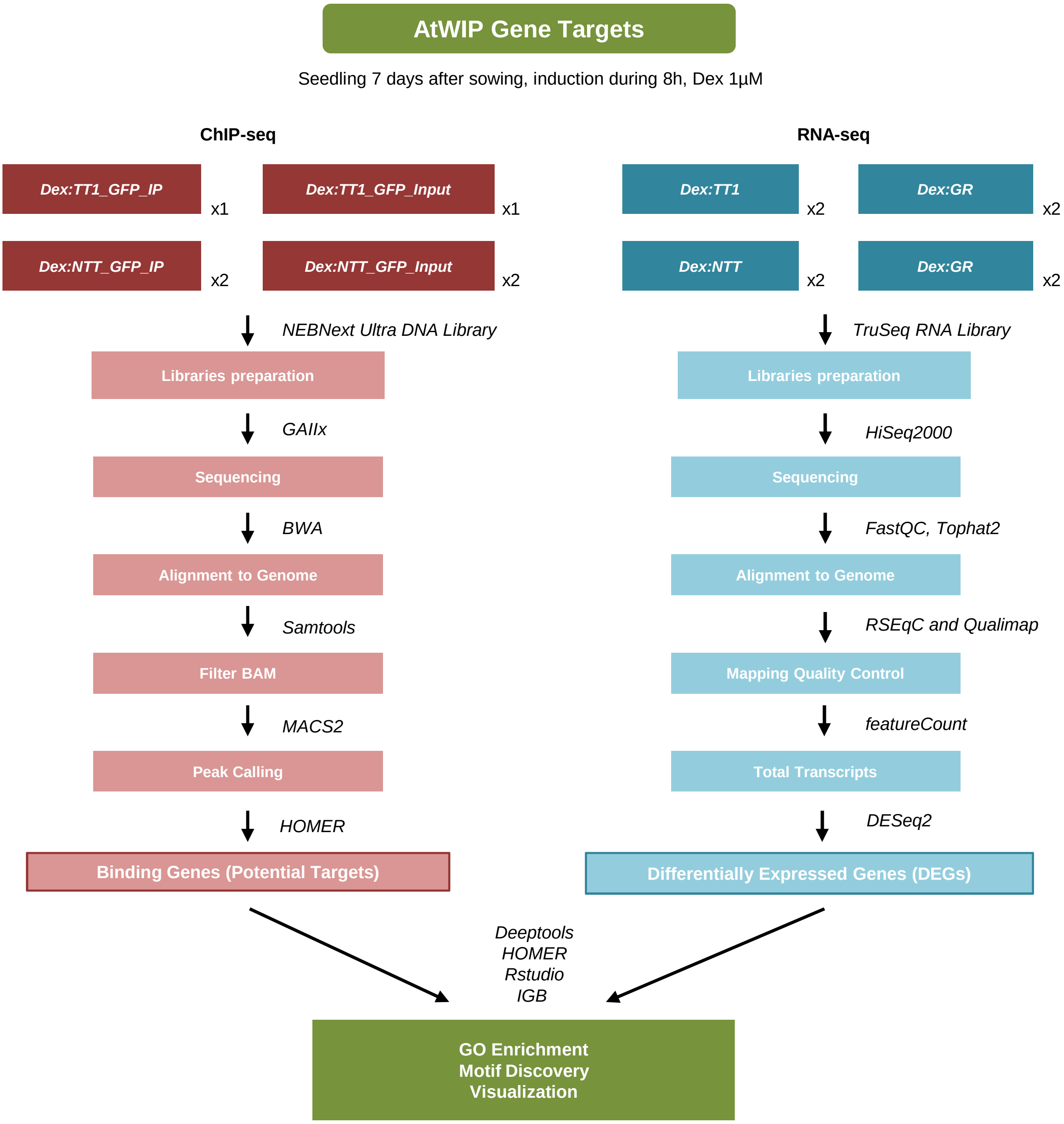

Supplementary Figure 7. Workflow of ChIP-seq and RNA-seq samples preparation and data analysis.

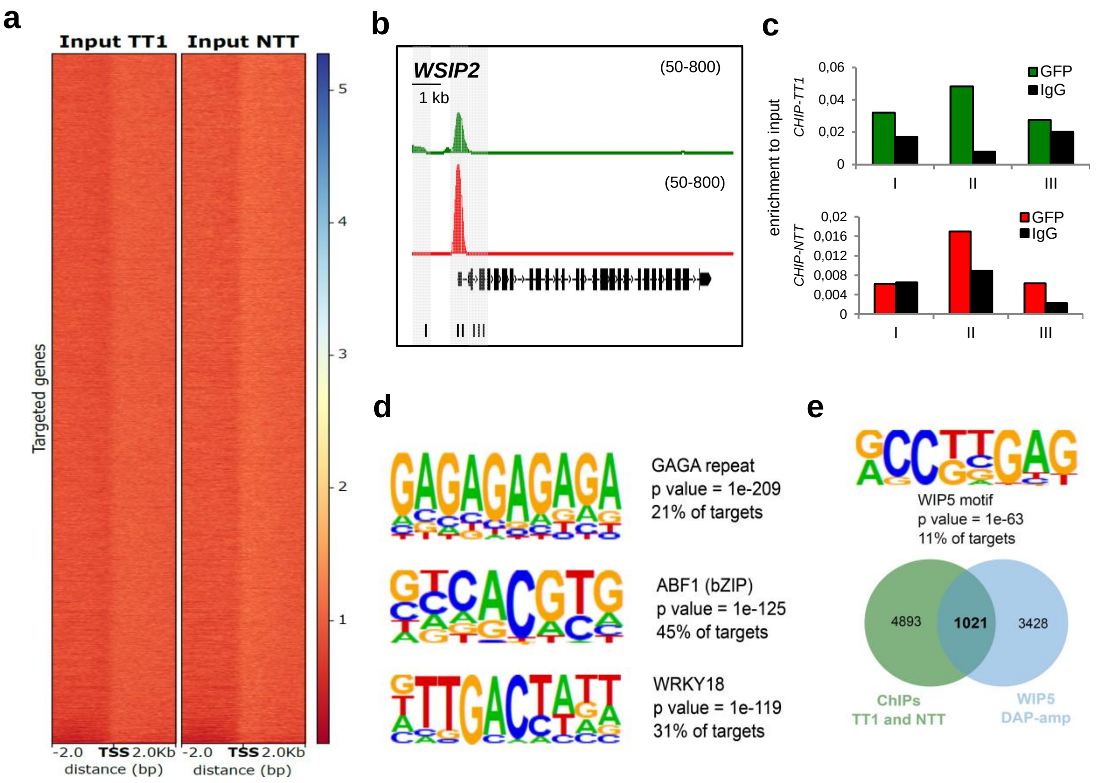

Supplementary Figure 8. Validation of WIP ChIP-seq experiments a) Input of *TT1-GFP* and *NTT-GFP* ChIP-seq analysis showing same binding region of genes from Figure **3a**. **b**) Binding peaks displayed by Integrated Genome Browser (IBG) software on *TT1-GFP* (green) and *NTT-GFP* (red) ChIP-seq data. **c**) ChIP-qPCR analysis to calculate the percentage of GFP and IgG antibodies enrichments in binding region of *WSPI2 TT1-GFP* and *NTT-GFP* immunoprecipitated chromatin. (*WSIP2=WUS-INTERACTING PROTEIN 2*). **d**) Binding motif found with HOMER on common TT1 and NTT ChIP targets. **e**) WIP5 binding motif found on TT1 and NTT common binding peaks. Comparison with WIP5 bound genes identified by O'Malley *et al*. (2017) using DNA affinity purification sequencing (DAP-seq) experiments.

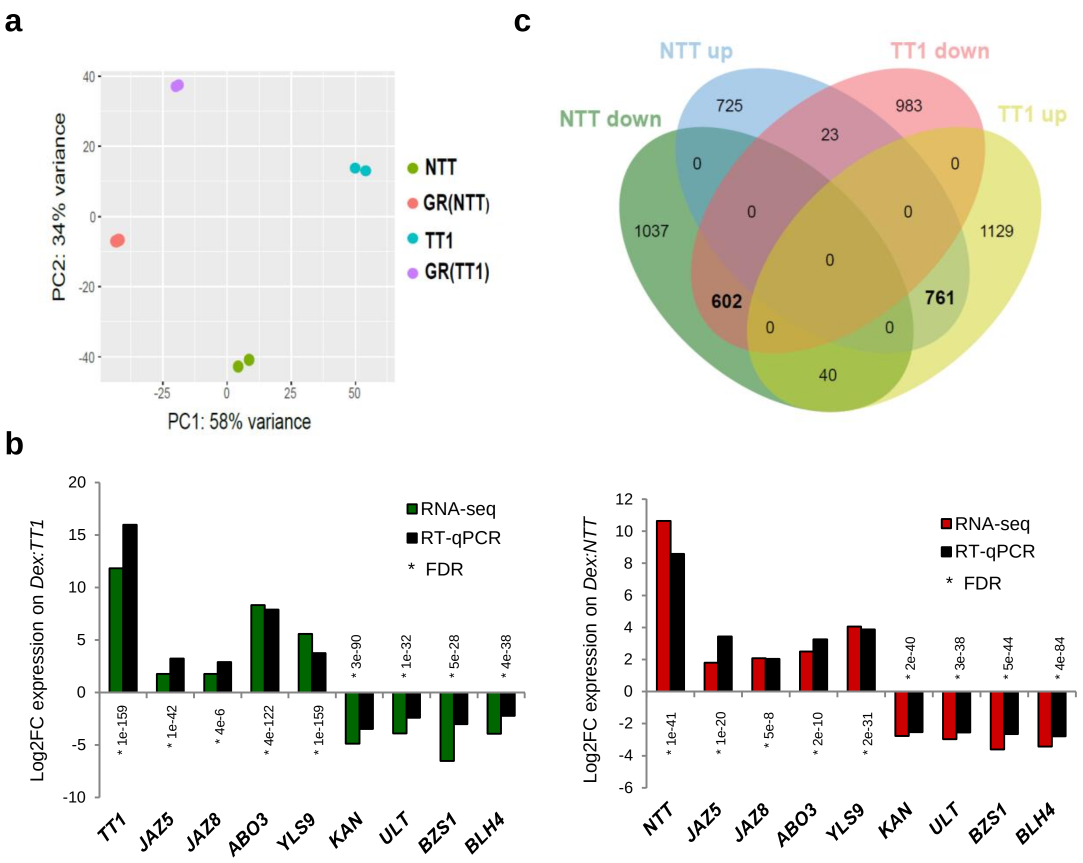

Supplementary Figure 9. RNA-seq results from *Dex:TT1* and *Dex:NTT* induced lines. **a**) Principal component analysis (PCA) of gene expression levels in *Dex:TT1* and *Dex:NTT* transgenic plants that had been induced with 1µM Dex. *Dex:GR* control for each line was analyzed separately. **b**) Quantitative PCR (RT-qPCR) confirmation of the RNA-sequencing (RNA-Seq) expression profiles of 8 differentially expressed genes on *Dex:TT1* and *Dex:NTT* samples. Log2 fold change (FC) was calculated relative to corresponding *Dex:GR* samples. *False Discovery Rate* (*FDR*) calculated from RNA-seq data for each gene is indicated with an asterisk (*). Actin was used as a reference gene for normalization. **c**) The Venn diagram shows the differentially (DEGs) expressed genes shared by TT1 and NTT. Highlighted numbers represent down (602) and up (761) regulated common direct target genes.

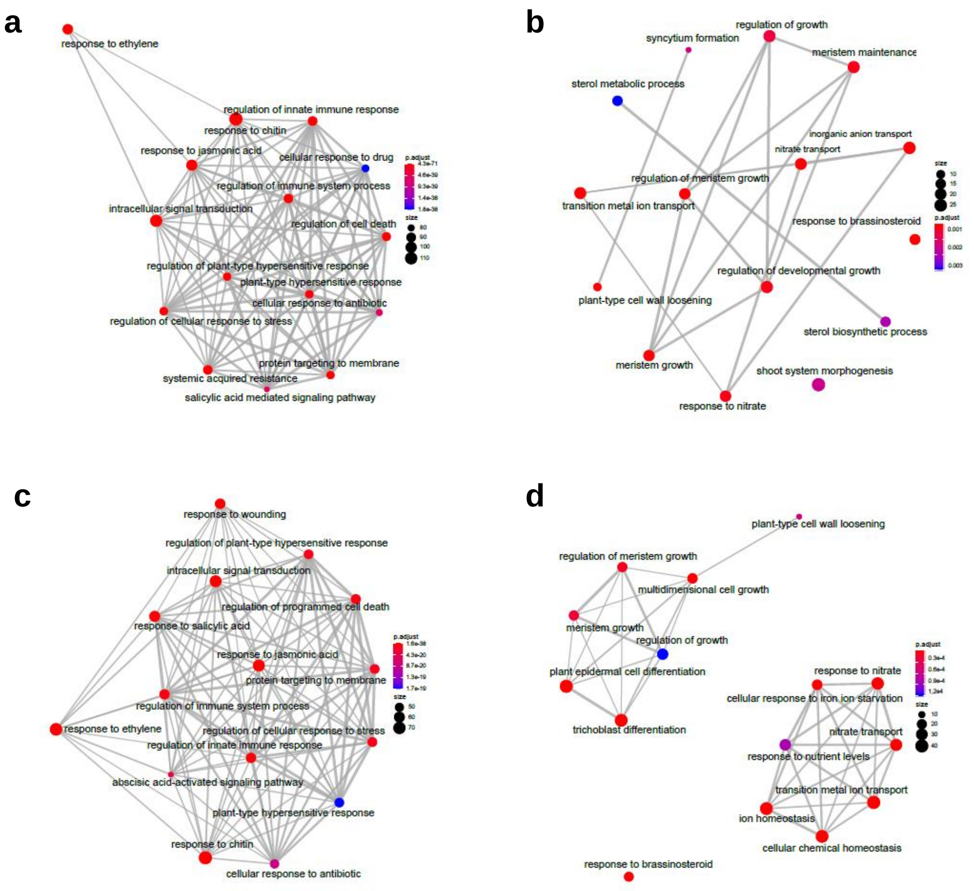

Supplementary Figure 10. Interact graphs of enriched GO terms in TT1 and NTT direct target genes. GO terms were selected and applied to Cytoscape software to generate GO interact graphs for **a**) UP and **b**) DOWN regulated target genes on TT1 data, **c**) UP and **d**) DOWN regulated target genes on NTT data. Colors from red to blue indicate *p* value from low to high. Thickness of lines indicates the relative strength of the association between two GO terms

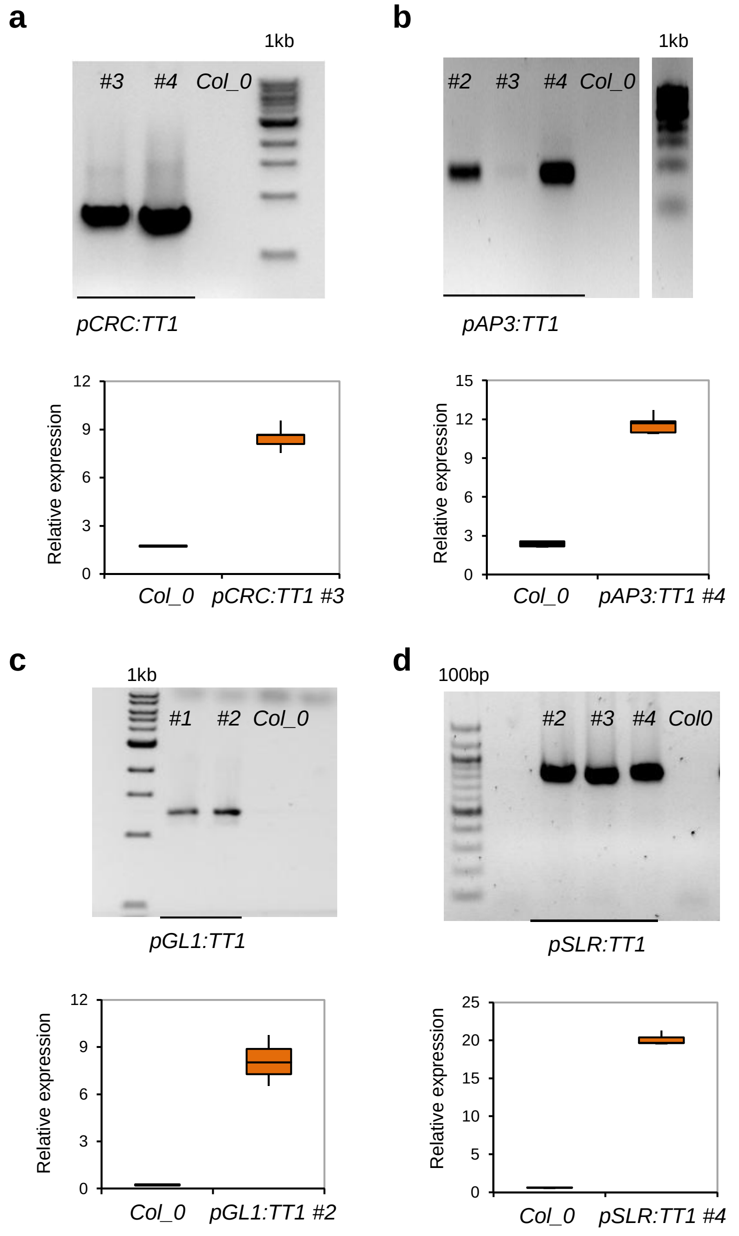

Supplementary Figure 11. Transgenic lines expressing *TT1* under the control of different tissue-specific promoters. T-DNA validation detected by PCR and quantification of *TT1* expression by RT-qPCR on reproductive organs: carpel specific promoter, *CRABS CRAW, pCRC* (**a**); petal and stamen specific promoter, *APETALA, pAP3* (**b**); and on vegetative tissues: trichome specific promotor, *GLABRA1*, *pGL1* (**c**), lateral roots specific promotor, *SOLITARY ROOTS*, *pSLR* (**d**). Corresponding primers are listed on Table S5.

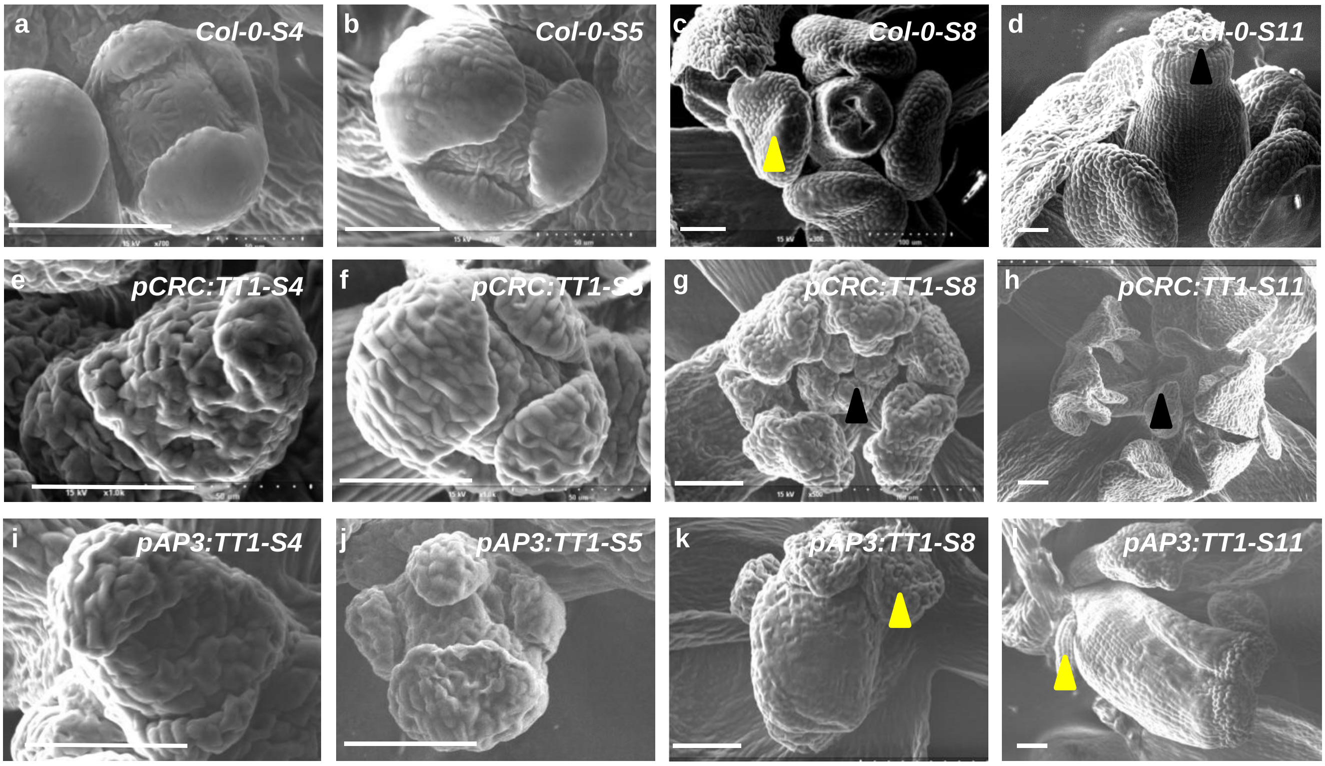

Supplementary Figure 12. Electronic microscopy on flowers of *Col-0* (a-d), *pCRC:TT1* (e-h) *and AP3:TT1* (i-l) transgenic lines. Different stages of flower development were observed (S4, S5, S8 and S11). In *pCRC:TT1* lines development of carpel has been severely affected (black head arrow), whereas in *pAP3:TT1* development of petals and stamens are stopped (yellow head arrowheads). Scale = 50µm.
