# Supplementary material for: Integrative genome-wide analysis reveals the role of WIP proteins in inhibition of growth and development
Source: Commun Biol. 2020 May 15;3:239. doi: 10.1038/s42003-020-0969-2 (PMC7229033; doi:10.1038/s42003-020-0969-2)
Supplement: Supplementary file 7 — Reporting summary [file 42003_2020_969_MOESM7_ESM.pdf]

nature research

Corresponding author(s): Bendahmane, COMMSBIO-20-0222

Last updated by author(s): 2020/03/10

Reporting Summary

Nature Research wishes to improve the reproducibility of the work that we publish. This form provides structure for consistency and transparency in reporting. For further information on Nature Research policies, see Authors & Referees and the Editorial Policy Checklist.

Please do not complete any field with "not applicable" or n/a. Refer to the help text for what text to use if an item is not relevant to your study.
For final submission: please carefully check your responses for accuracy; you will not be able to make changes later.

Statistics

For all statistical analyses, confirm that the following items are present in the figure legend, table legend, main text, or Methods section.

n/a	Confirmed	
☐	☒	The exact sample size (*n*) for each experimental group/condition, given as a discrete number and unit of measurement
☐	☒	A statement on whether measurements were taken from distinct samples or whether the same sample was measured repeatedly
☐	☒	The statistical test(s) used AND whether they are one- or two-sided<br>*Only common tests should be described solely by name; describe more complex techniques in the Methods section.*
☒	☐	A description of all covariates tested
☐	☒	A description of any assumptions or corrections, such as tests of normality and adjustment for multiple comparisons
☒	☐	A full description of the statistical parameters including central tendency (e.g. means) or other basic estimates (e.g. regression coefficient) AND variation (e.g. standard deviation) or associated estimates of uncertainty (e.g. confidence intervals)
☐	☒	For null hypothesis testing, the test statistic (e.g. *F*, *t*, *r*) with confidence intervals, effect sizes, degrees of freedom and *P* value noted<br>*Give P values as exact values whenever suitable.*
☒	☐	For Bayesian analysis, information on the choice of priors and Markov chain Monte Carlo settings
☒	☐	For hierarchical and complex designs, identification of the appropriate level for tests and full reporting of outcomes
☒	☐	Estimates of effect sizes (e.g. Cohen's *d*, Pearson's *r*), indicating how they were calculated

Our web collection on statistics for biologists contains articles on many of the points above.

Software and code

Policy information about availability of computer code

Data collection	No special software was used for data collection
Data analysis	CHIP-seq<br>Reads were mapped with BWA (Burrows-Wheeler Aligner) onto Arabidopsis genome release from Ensembl database, then only reads with mapQ > 30 and uniquely mapped were kept using Samtools (http://www.htslib.org/).<br>The MACS2 software was used to identify significantly enriched regions (q-value <10e-10)38. Visualization and analysis of genome-wide enrichment profiles were done with IGB (Integrated Genome Browser, https://bioviz.org/). Peak annotations were assigned using HOMER, keeping only peaks in an interval of [-1500; + 150] around the TSS (Transcription Start Site).<br><br>RNA-seq<br>Quality was assessed using FastQC (version 0.11). Tophat2 (version 2.1.0) has been used to generate the mapping files and RSeQC aligned reads above 94% of read pairs of each sample correctly to TAIR (version 10). The mapped reads were assigned to genes with featureCount (v1.5.0-p3). DESeq2 (version 1.10.1) analysis was employed for differentially expressed gene calling (p-value adjusted > 0.05 and log2 FC <-2 and >2).

For manuscripts utilizing custom algorithms or software that are central to the research but not yet described in published literature, software must be made available to editors/reviewers. We strongly encourage code deposition in a community repository (e.g. GitHub). See the Nature Research guidelines for submitting code & software for further information.

Data

Policy information about availability of data

All manuscripts must include a data availability statement. This statement should provide the following information, where applicable:

- Accession codes, unique identifiers, or web links for publicly available datasets
- A list of figures that have associated raw data
- A description of any restrictions on data availability

The raw RNA-seq and ChIP-seq data described in this study have been deposited to the NCBI Short Read Archive (SRA) database under the BioProject ID PRJNA608903.

Field-specific reporting

Please select the one below that is the best fit for your research. If you are not sure, read the appropriate sections before making your selection.

☒ Life sciences ☐ Behavioural & social sciences ☐ Ecological, evolutionary & environmental sciences

Life sciences study design

All studies must disclose on these points even when the disclosure is negative.

Sample size	For CHIP-seq experiments, seven-days old seedlings (pool of aprox. 100 seedlings per sample) of Dex:TT1-GFP and Dex:NTT-GFP lines were transferred to MS liquid medium supplied with 1µM Dex for 8h.<br>For RNA-seq, seven-days old seedlings of Dex:TT1, Dex:NTT and corresponding Dex:GR lines were treated with Dex (1µM during 8h).
Data exclusions	No data were excluded from analysis, all data produced are available.
Replication	For the ChIP-seq experiments, we performed two biological replicate for Dex:NTT-GFP and one biological replicate for De:TT1-GFP.Two biological replicates were collected from each genotype for RNA-seq experiments (in total eight libraries).
Randomization	Seedlings were grown in plates and organized in a randomized manner in the plant growth incubator.
Blinding	Blinding was not relevant to this study.

Reporting for specific materials, systems and methods

We require information from authors about some types of materials, experimental systems and methods used in many studies. Here, indicate whether each material, system or method listed is relevant to your study. If you are not sure if a list item applies to your research, read the appropriate section before selecting a response.

Materials & experimental systems

n/a	Involved in the study	
☐	☒	Antibodies
☒	☐	Eukaryotic cell lines
☒	☐	Palaeontology
☒	☐	Animals and other organisms
☒	☐	Human research participants
☒	☐	Clinical data

Methods

n/a	Involved in the study	
☐	☒	ChIP-seq
☒	☐	Flow cytometry
☒	☐	MRI-based neuroimaging

Antibodies

Antibodies used	anti-GFP (Clontech 632592) and anti-IgG antibody (Millipore) antibodies
Validation	Specificity of the antibodies has been tested by the supplier.

ChIP-seq

Data deposition

☒ Confirm that both raw and final processed data have been deposited in a public database such as GEO.

☒ Confirm that you have deposited or provided access to graph files (e.g. BED files) for the called peaks.

Data access links<br>*May remain private before publication.*	The raw RNA-seq and ChIP-seq data described in this study have been deposited to the NCBI Short Read Archive (SRA) database under the BioProject ID PRJNA608903.<br>https://dataview.ncbi.nlm.nih.gov/object/PRJNA608903?reviewer=53fqkm1t1fpo118k47gf27a48o
Files in database submission	ChipSeq of Arabidopsis thaliana Dex:TT1_GFP Dex:TT1_GFP_chip DDLS25.fastq.gz<br>Input of Arabidopsis thaliana Dex:TT1_GFP Dex:TT1_GFP_input DDLS22.fastq.gz<br>ChipSeq of Arabidopsis thaliana Dex:NTT_GFP rep1 Dex:NTT_GFP_chip_r1 TDJG66.fastq.gz<br>Input of Arabidopsis thaliana Dex:NTT_GFP rep1 Dex:NTT_GFP_chip_input_r1 TDJG65.fastq.gz<br>ChipSeq of Arabidopsis thaliana Dex:NTT_GFP rep2 Dex:NTT_GFP_chip_r2 TDJG69.fastq.gz<br>Input of Arabidopsis thaliana Dex:NTT_GFP rep2 Dex:NTT_GFP_chip_input_r2 TDJG64.fastq.gz
Genome browser session<br>(e.g. UCSC)	NA

Methodology

Replicates	For the ChIP-seq experiments, we performed two biological replicate for Dex:NTT-GFP and one biological replicate for De:TT1-GFP.
Sequencing depth	The average read number for each single replicate is ca. 29M, of which 39-76% unique mapping. Read length: 50bp in single-end.
Antibodies	anti-GFP (Clontech 632592)
Peak calling parameters	MACS2 (2.1.2) software was used to identify significantly enriched regions (q-value <10e-10)38. Peak annotations were assigned using HOMER, keeping only peaks in an interval of [-1500; + 150] around the TSS.<br>Peaks were called using the following command:<br>macs2 callpeak -t DDLS25.filt.nodup.srt.bam -c DDLS22.filt.nodup.srt.bam -f AUTO -g 119146348 --outdir TT1 -n TT1 -q 10e-10
Data quality	In our analysis, we used peaks at FDR < 1%, and > 25% and >67% of the TT1 and NTT peaks, respectively, were above 5-fold enrichment. Reads identified as PCR duplicates were discarded using Picard Toolkit (https://broadinstitute.github.io/picard/).
Software	Heatmaps were drawn using Deeptools software; hexbin plot with R and ggplot2 library. ChIP-seq data signal tracks were visualized with IGB (Integrated Genome Browser, https://bioviz.org/).

This checklist template is licensed under a Creative Commons Attribution 4.0 International License, which permits use, sharing, adaptation, distribution and reproduction in any medium or format, as long as you give appropriate credit to the original author(s) and the source, provide a link to the Creative Commons license, and indicate if changes were made. The images or other third party material in this article are included in the article's Creative Commons license, unless indicated otherwise in a credit line to the material. If material is not included in the article's Creative Commons license and your intended use is not permitted by statutory regulation or exceeds the permitted use, you will need to obtain permission directly from the copyright holder. To view a copy of this license, visit http://creativecommons.org/licenses/by/4.0/
