# Supplementary material for: Integrative genome-wide analysis reveals the role of WIP proteins in inhibition of growth and development
Source: Commun Biol. 2020 May 15;3:239. doi: 10.1038/s42003-020-0969-2 (PMC7229033; doi:10.1038/s42003-020-0969-2)
Supplement: Supplementary file 9 — Description of Additional Supplementary Files [file 42003_2020_969_MOESM9_ESM.pdf]

Supplementary Data 1. WIP related genomic regions identified by ChIP-Seq experiments. Genomic regions identified as commonly enriched in the *Dex:TT1* and *Dex:NTT* ChIP-Seq experiments. Genomic region coordinates (start and end of peaks, according to the TAIR genome release 10), as well as peak score, distance to TSS and peak position are listed.

Supplementary Data 2. Differentially enriched genes (DEGs) identified by RNA-seq. Differentially enriched genes (DEGs) found on *Dex:TT1* and *Dex:NTT* transgenic lines compared to *Dex:GR* control lines (FDR < 0.05, FC log2 ± 2.0) identified by RNA-seq.

Supplementary Data 3. Putative direct target genes of WIP proteins. Putative direct target genes of TT1 and NTT identified by combination of ChIP-Seq and RNA-seq data.

Supplementary Data 4. Enriched GO terms associated with the WIP function. GO term analyses identified in putative direct target of TT1 and NTT using Rstudio.

Supplementary Data 5. List of primers. Primers used for cloning, RT-qPCR and ChIP-qPCR analyses.
